# Trigeminocervical pain sensitivity during the migraine cycle depends on headache frequency

**DOI:** 10.1007/s10072-023-06858-x

**Published:** 2023-06-13

**Authors:** Stefano Di Antonio, Lars Arendt-Nielsen, Marta Ponzano, Francesca Bovis, Paola Torelli, Cinzia Finocchi, Matteo Castaldo

**Affiliations:** 1 Department of Health Science and Technology, Center for Pain and Neuroplasticity (CNAP), SMI, School of Medicine, Aalborg University, Aalborg, Denmark; 2 Department of Neuroscience, Rehabilitation, Ophthalmology, Genetics and Maternal Child Health, Genoa, Italy; 3 Department of Medical Gastroenterology, Mech-Sense, Aalborg University Hospital, Aalborg, Denmark; 4 Steno Diabetes Center North Denmark, Clinical Institute, Aalborg University Hospital, 9000 Aalborg, DK Denmark; 5 Department of Health Sciences (DISSAL), Section of Biostatistics, University of Genoa, Genoa, Italy; 6 Headache Centre, Department of Medicine and Surgery, University of Parma, Parma, Italy; 7 Ospedale San Paolo, ASL 2 Savonese, 17100 Savona, Italy

**Keywords:** Low frequency episodic migraine, High frequency episodic migraine, Chronic migraine, Migraine cycle, Quantitative sensory testing, Pain sensitivity

## Abstract

**Objective:**

This experimental study aimed to assess pain sensitivity in low-frequency episodic migraine (LFEM), high-frequency episodic migraine (HFEM), and chronic migraine (CM) patients across the different phases of the migraine cycle.

**Method:**

In this observational, experimental study, clinical characteristics (diary and time from the last/next headache attack), and quantitative sensory testing (QST) (wind-up pain ratio (WUR) and pressure pain threshold (PPT) from the trigeminal area and PPT from the cervical spine) was performed. LFEM, HFEM, and CM were assessed in each of the 4 migraine phases (HFEM and LFEM: interictal, preictal, ictal, and postictal; CM: interictal and ictal) and compared vs. each other’s (matched for the phase) and controls.

**Results:**

A total of 56 controls, 105 LFEM, 74 HFEM, and 32 CM were included. No differences in QST parameters were observed between LFEM, HFEM, and CM in any of the phases.

During the interictal phase and when comparing with controls the following were found: 1) LFEM had lower trigeminal PPT (*p* = 0.001) and 2) lower cervical PPT (*p* = 0.001). No differences were observed between HFEM or CM and healthy controls. During the ictal phase and when comparing with controls the following were found: HFEM and CM had 1) lower trigeminal PPTs (HFEM *p* = 0.001; CM = *p* < 0.001), 2) lower cervical PPT s (HFEM *p* = 0.007; CM *p* < 0.001), and 3) higher trigeminal WUR (HFEM *p* = 0.001, CM *p* = 0.006). No differences were observed between LFEM and healthy controls. During the preictal phase and when comparing with controls the following were found: 1) LFEM had lower cervical PPT (*p* = 0.007), 2) HFEM had lower trigeminal (*p* = 0.013) and 3) HFEM had lower cervical (*p* = .006) PPTs. During the postictal phase and when comparing with controls the following were found: 1) LFEM had lower cervical PPT (*p* = 0.003), 2) HFEM had lower trigeminal PPT (*p* = 0.005), and 3) and HFEM had lower cervical (*p* = 0.007) PPTs.

**Conclusion:**

This study suggested that HFEM patients have a sensory profile matching CM better than LFEM. When assessing pain sensitivity in migraine populations, the phase with respects to headache attacks is of utmost importance and can explain the inconsistency in pain sensitivity data reported in the literature.

**Supplementary Information:**

The online version contains supplementary material available at 10.1007/s10072-023-06858-x.

## Introduction

Migraine is a complex brain disorder characterized by cyclic changes in experimentally assessed pain sensitivity [1]. Migraine is considered among the primary causes of disability worldwide and the first cause of disability under the age of fifty [2, 3]. The International Classification of Headache Disorders (ICHD) differentiates migraine into chronic (15 or more monthly headache days) and episodic (less than 15 monthly headache days) [4]. However, high-frequency episodic migraine (HFEM, 8 or more monthly headache days) could be considered as disabling as chronic migraine (CM) [5, 6]. Thus, some authors proposed that HFEM should be differentiated from low-frequency episodic migraine (LFEM, less than 8 monthly headache days) and ﻿included in a revised diagnostic criterion for CM[5]. One of the possible mechanisms associated with migraine chronification is enhanced pain sensitivity [7], and this hypothesis is supported by data suggesting that HFEM and CM had more symptoms related to pain sensitization and higher prevalence of allodynia compared to LFEM, without differences between HFEM and CM [8, 9]. However, as these studies assessed pain sensitization using questionnaires, additional quantitative evidence supporting that HFEM patients are more similar to CM than LFEM is needed.

Quantitative sensory testing (QST) has been used as a proxy to assess pain sensitivity and hence pain sensitization in patients with migraine [10]. To the author’s knowledge, only one research group compared QST values between LFEM and HFEM patients [11–13]. So far, no study has assessed differences in QST between LFEM, HFEM, and CM. Even if no differences were observed between LFEM and HFEM interictally [11–13], patients should be assessed in all migraine phases to fully understand the differences/similarities between the two groups as pain sensitivity changes during the migraine cycle [14–16].

Thus, to fulfill this gap in the literature, this study aimed to 1) assess if pain sensitivity (QST) differed across LFEM, HFEM, and CM patients assessed during different phases of the migraine cycle, and 2) assess if pain sensitivity correlates with time from the headache attack and if this differed in LFEM, HFEM, and CM across the migraine cycle. We hypothesize that 1) HFEM patients would have a pain sensitivity profile matching CM more than LFEM, and 2) pain sensitivity would correlate with time from the headache attack in HFEM and CM, but not in LFEM.

A better understanding of similarities and differences in pain sensitivity across the different migraine phases in migraine patients with different headache frequencies may help phenotyping patients and provide knowledge which at some stage may be utilized in individualized treatment approaches.

## Method

### Design

This multicenter, cross-sectional, observational study was conducted in the Headache Center of Parma and Genova (Italy) and approved by the Ligurian (244/2018) and “Area Vasta Emilia-Nord” (18,305/2019) regional ethic committee. All subjects signed an informed consent form and were assessed between April 2019 and February 2022.

## Population

Patients on waiting lists to receive their first visit to the Headache Center were invited to participate in this study. Men and women aged between 18 and 65 with migraine for at least 3 months were included. Patients were excluded if they had: any other primary/secondary headache; less than 1 headache attack in four weeks; changes of headache characteristics, or onset of a “new” headache after COVID-19 infection/vaccination; any other neurologic, psychiatric, rheumatologic (i.e., fibromyalgia, rheumatoid arthritis) or systemic pathology with medical diagnosis; history of head/neck trauma in the previous year; received cervical/head surgery; received manual therapy in the cervical spine, cervical anesthetic block, or botulin injection in the last 6 months; changed the prophylactic treatment in the last 3 months; were unable to speak and understand Italian; patients with headache attack modified by acute pharmacologic treatment in the previous 24 h who developed a headache in the 24 h after the assessment (criterion adopted to avoid including ictal patients in which the current attack was transitorily modified by acute medication).

Control participants were recruited specifically for this study. They were defined as healthy subjects with a maximum of two headache episodes per year that did not fulfill the criteria for migraine or any other primary headache type with no family history of migraine or other primary headaches. The inclusion criteria for the control subjects were the same as the criteria used for migraine patients.

## Procedure

**Fig. 1:**
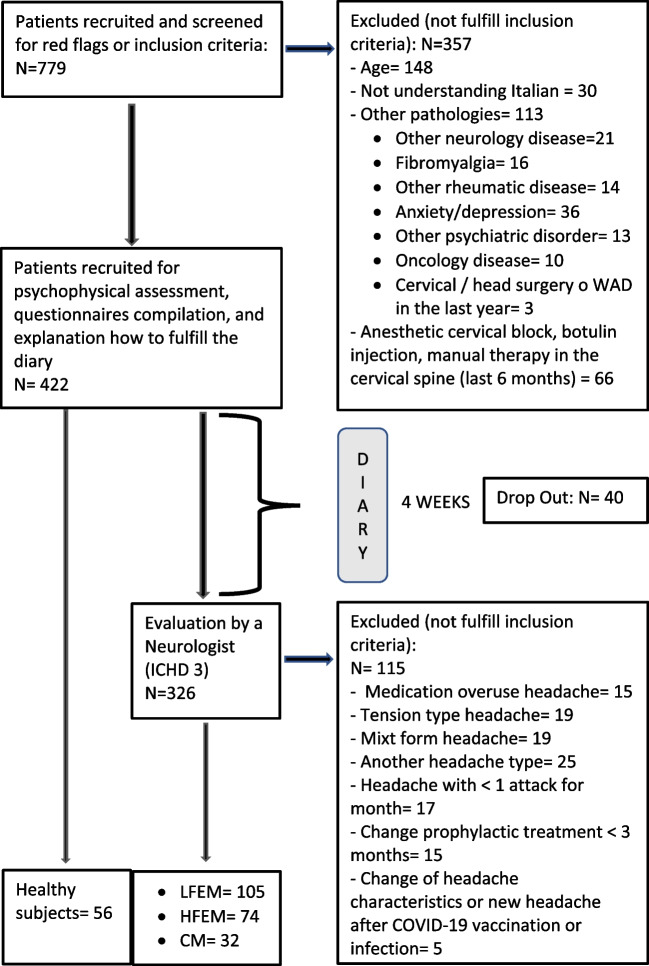
Flow chart. CM: chronic migraine; HFEM: high frequency episodic migraine; LFEM: low frequency episodic migraine; ICHD: international classification headache disorder; N: number;

The first screening was made by telephone interview where patients were excluded if they presented any signs of red flags [17] or any exclusion criteria. Healthy controls were recruited from university students, hospital staff and university staff, and the general population through print and social media advertising. During the examination, one physiotherapist for each recruitment center (S.D., M.C.), blinded to the subject’s diagnosis, performed the assessment (QST examination and explanation of how to fulfill a diary for the following four weeks) and recorded the interval between the assessment and the last headache attack. To maintain the blindness of the assessor, the QST examination was performed before asking any questions regarding headache phases and explaining how to fulfill the diary. Four weeks following the first evaluation, patients were visited by a neurologist who performed a diagnosis of headache according to the ICHD-3 [4]. Migraine patients with or without aura were divided into three subgroups according to headache frequency (Fig. 1) [5, 9].

- Low-frequency episodic migraine (LFEM): patients with less than 8 headache days in a month.
- High-frequency episodic migraine (HFEM): patients with 8 or more headache days in a month
- Chronic Migraine (CM): patients with 15 or more headache days in a month (at least 8 or more headache days had to fulfill the criteria for migraine)

Then, the neurologist retrospectively assessed the diary and recorded the interval between the first assessment and the following headache attack. LFEM, HFEM, and CM patients were categorized according to the phase of the migraine cycle in which the first examination was performed [4, 18]:

LFEM and HFEM were categorized in:

- Interictal phase: no headache attack occurred in the 48 h before or after the evaluation.
- Preictal phase: headache attack occurred in the 48 h after the evaluation.
- Ictal phase: headache attack during the evaluation.
- Postictal phase: headache attack occurred in the 48 h before the evaluation.

LFEM and HFEM patients with a headache attack that occurred in the 48 h after and in the 48 h before the evaluation were categorized both in the preictal and the postictal phases.

CM patients were categorized in:

- Interictal phase: no headache during the evaluation.
- Ictal phase: headache attack during the evaluation.

Due to the shorter period occurring between two consecutive migraine attacks in CM patients, it was not possible to identify a preictal and a postictal phase in this subgroup of patients.

## Assessments

General and clinical characteristics were assessed for each patient. Patients used a daily updated diary recording the total use of drugs and the frequency, intensity, and duration of headache attacks (Table 1).

**Table 1:** clinical and general characteristics

		CM (32)		LFEM (105)				HFEM (74)			
	Controls (56)	Interictal (14)	Ictal (18)	Interictal (41)	Preictal (33*)	Ictal (17)	Postictal (21*)	Interictal (14)	Preictal (27**)	Ictal (23)	Postictal (20**)
Age, mean (SD)	37.2(14.3)	35.2(10.0)	40.8(10.3)	36.2(11.4)	37.6(12.8)	42.4(11.0)	38.4(12.8)	40.2(12.0)	41.7(14.3)	36.4(11.3)	36.4(12.4)
Gender, N (%)											
Female	40(71%)	12(86%)	16(89%)	32(78%)	25(76%)	14(82%)	14(67%)	8(57%)	21(78%)	22(96%)	16(80%)
Male	16(29%)	2(14%)	2(11%)	9(22%)	8(24%)	3(18%)	4(33%)	6(43%)	6(22%)	1(4%)	4(20%)
BMI, mean (SD)	22.1(2.7)	21.7(3.6)	25.0(4.2)	23(4.4)	23.2(3.3)	23.4(3.7)	23.4(3.8)	22.6(3.1)	22.4(2.7)	23.5(5.1)	22.8(2.4)
Use of preventive pharmacological therapy, N (%)											
Yes	0(0%)	5(36%)	7(39%)	4(10%)	6(18%)	0(0%)	20(95%)	1(7%)	4(15%)	2(9%)	18(90%)
No	56(100%)	9(64%)	11(61%)	37(90%)	27(82%)	17(100%)	1(5%)	13(93%)	23(85%)	21(91%)	2(10%)
Acute treatment 24 h Before the evaluation, N(%)											
Yes	1(2%)	2(14%)	5(28%)	3(7%)	4(12%)	2(12%)	8(38%)	1(7%)	4(15%)	8(35%)	7(35%)
No	55(98%)	12(86%)	13(72%)	38(93%)	29(88%)	15(88%)	13(62%)	13(93%)	23(85%)	15(65%)	13(65%
Frequency, mean day/ four weeks (SD)	………	19.1(4.2)	21.2(4.8)	4.1(1.8)	4.8(1.5)	5.1(1.6)	4.3(1.7)	10.4(1.2)	10.2(1.7)	10.4(1.8)	10.6(1.9)
Duration, mean hours/day (SD)	………	10.5(5.6)	9.9(4.5)	8(5.8)	5.7(2.9)	8.3(3.5)	7.7(3.3)	5.6(3)	7.7(5.9)	6.4(3.3)	7.3(5.3)
Intensity, mean NPRS 0–10 (SD)	………	5.2(1.5)	5.6(1.8)	5.7(1.8)	5.4(1.2)	4.6(2.0)	5.4(1.6)	5.6(1.5)	5.5(2.3)	5.7(1.7)	6.2(2.0)
Drugs, mean number of tablets / four weeks (SD)	………	10.4(5.2)	10.0(8.7)	2.9(4.4)	3.8(2.9)	2.7(2.2)	4.5(4.5)	8.9(4.5)	6.7(4.2)	6.7(4.8)	8.0(5.0)
Time from last headache attack, mean hours (SD)	………	26.6(22.4)	0	260.9 (191.6)	185.2 (186.4)	0	25.6 (11.0)	138.1 (108.0)	107.9 (109.7)	0	20.8 (10.2)
Time to the next headache attack, mean hours (SD)	………	31.2(41.8)	0	225.3 (165.3)	20.2 (12.2)	0	151.1 (179.6)	61.9 (15.9)	17.8 (10.3)	0	61.0 (70.8)

BMI: body mass index; CM: chronic migraine; HFEM: high frequency episodic migraine; LFEM: low frequency episodic migraine; SD: standard deviation; N: number; NPRS: numeric pain rating scale; * 7 LFEM patients were included both in the preictal an postictal group; **10 HFEM patients were included both in the preictal an postictal group

### Quantitative sensory testing (QST)

QST was performed from distal pain-free areas first, then the cervical area, and finally the trigeminal area (symptomatic side in patients with unilateral migraine; dominant side in patients with side/shift or bilateral migraine and in controls) [18]. The following variables were assessed.

1. *Pressure pain threshold (PPT):* Pressure pain thresholds to hand-held algometry (Somedic AB, Sweden), probe area 1cm^2^, 30 kPa/s force increase were assessed over the trigeminal area (temporalis muscles), the upper cervical spine (sum of left and right articular pillars), and lower cervical spine (sum of left and right articular pillars). The lower the PPTs, the higher the sensitization. PPT has high reliability (test–retest reliability (TR-R) = 0.88; interobserver reliability (IO-R) = 0.84)[19].
  3. *Wind-up ratio (WUR):* the WUR assessed the temporal summation of mechanical pinprick pain (50.1 g pinprick) over the trigeminal area temporalis muscle). The subject gave a pain rating (11-point Numeric Rating Scale) for the first and last stimulus of 10 stimuli. The difference between the pain rating of the ten stimuli series and the pain rating of the first stimulus was calculated. The higher the WUR, the higher the sensitization. WUR exhibited good reliability (TR-R = 0.67; IO-R = 0.56) [19].

Details of the QST assessment were previously presented [9, 18, 20].

## Statistical analysis

Sample size calculation was performed using G*Power 3.1. The sample size needed to achieve a medium/large effect size (f^2^: 0.20) with an alpha level of 0.008 and the desired power of 80% in a two-tail linear regression model with 6 predictors was 107. The sample size needed to achieve a medium/large effect size (f^2^: 0.20) with an alpha level of 0.017 and the desired power of 80% in a two-tail linear regression model with 6 predictors was 94. As multiple analyses were performed using different samples, we included a total of 267 subjects assuring that at least 107 subjects were included in the analysis that compared Controls, LFEM, HFEM, and CM (Bonferroni corrected p-value of 0.008), and at least 94 subjects were included in the analysis that compared Controls, LFEM, and HFEM (Bonferroni corrected p-value of 0.017). The sample size needed to achieve a large effect size (r: 0.50) with an alpha level of 0.05 and the desired power of 80% in a correlation analysis was 26. As the correlation analysis was performed in each migraine subgroup (CM, HFEM, LFEM), at least 26 patients in each group were needed.

All data were presented as mean (standard deviation), median (interquartile range), or numbers (percentage). Data distribution was assessed using the Shapiro–Wilk test and non-normal data were transformed to fulfill normality assumption. A preplanned analysis was performed. For subjects included between August 2021 and February 2022, this was the primary analysis of these data. For subjects included between April 2019 and August 2021, this was a secondary analysis of these data and previous results were reported elsewhere[9, 18, 20].

To assess differences in QST values between LFEM, HFEM, CM, and controls, we performed a linear regression for each migraine phase using QST variables as the dependent variable and group as the predictor, while adjusting for possible confounders (gender, age, body mass index, use of preventive pharmacological therapy, and use of acute treatment in the 24 h before the evaluation). LFEM, HFEM, and CM patients were assessed separately in each migraine phase (LFEM and HFEM in the interictal, preictal, ictal, postictal phase; CM in the interictal and ictal phase) while in each model all the Controls were included. As multiple comparisons were performed, a Bonferroni-corrected p-value was adopted (0.05 divided for the number of between-group comparisons performed in each analysis).

Then, to assess if the correlation between QST variables and time from the last or to the next headache attack differed in LFEM, HFEM, and CM, a Spearman correlations analysis was performed between QST results and time relative to the last or the next migraine attack in HFEM, LFEM, and CM (ictal and interictal) separately. When assessing the correlation between QST results and time relative to the last migraine attack, only LFEM and HFEM in the interictal, ictal, and postictal phase were analyzed. When assessing the correlation between QST results and time relative to the next migraine attack, only LFEM and HFEM in the interictal, ictal, and preictal phase were analyses. For all the correlation analyses, patients with a headache attack that occurred in the 48 h after and in the 48 h before the evaluation were excluded. The threshold accepted for the statistical significance of the correlation analyses was *p* < 0.05.

Patients with any missing data were excluded from the analysis. All tests of statistical significance were two-tailed and statistical analyses were performed using the SPSS software (version 24).

## Result

After 779 subjects were initially recruited, 267 were included (56 controls, 32 CM, 105 LFEM, and 74 HFEM) (Fig. 1). No patients were excluded due to missing data. General characteristics, clinical characteristics, and the phase in which the assessment occurred are shown in Table 1. All QST data were log-transformed to fulfill the normality assumption.

**Fig. 2:**
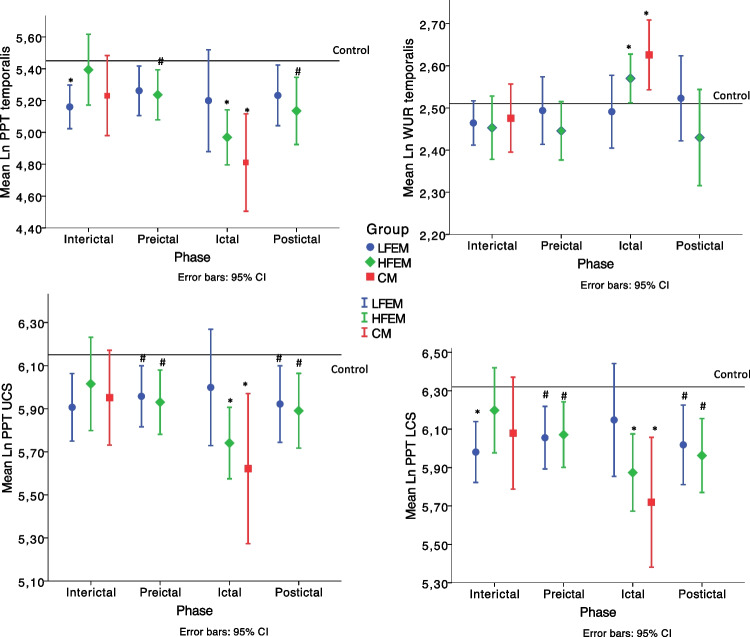
differences in QST values. CM: chronic migraine; HFEM: high-frequency episodic migraine; LFEM: low-frequency episodic migraine; LN: natural logarithm; MPT: mechanical pain threshold; PPT: pressure pain threshold; UCS: upper cervical spine; WUR: Wind-up ratio; kPa: kilopascal; g: gram; Reference lines represent mean value of Control. CM patients were included only in the interictal and ictal phases, but not in the preictal and postictal phases * Difference at *p* < 0.008 vs healthy controls. # Difference at *p* < 0.017 vs ictal CM

No differences in any QST parameters were observed between LFEM, HFEM, and CM, in all the phases (Fig. 2, Tables 2 and 3).

**Table 2:** Linear regression model using QST variable as the dependent variable, group as predictor and gender, age, body mass index, use of preventive pharmacological therapy, and use of acute treatment in the 24 h before the evaluation as covariate in interictal and ictal phases

Interictal phase (n = 115)	LFEM	HFEM	CM
PPT trigeminal, median (25^th^,75^th^) †			
LFEM: 171.8(133.6–240.8) kPa	Ref		
HFEM: 231.3(158.0–305.7) kPa	B = 0.1; *p* = 0.225	Ref	
CM: 193.4(129.8–262.9) kPa	B = -0.1; *p* = 0.621	B = -0.1; *p* = 0.580	Ref
CONTROLS 237.6(180.4–293.3) kPa	**B = 0.3;** ***p*** = 0.001*	B = 0.1; *p* = 0.289	B = 0.2; *p* = 0.103
WUR trigeminal, median (25^th^,75^th^) †			
LFEM: 2.0(0.0–3.5)	Ref		
HFEM: 1.5(1.0–2.3)	B < -0.1; *p* = 0.778	Ref	
CM 1.0(0.0–2.3)	B = -0.1; *p* = 0.370	B < -0.1; *p* = 0.610	Ref
CONTROLS 2.0(0.0–3.0)	B = -0.1; *p* = 0.184	B < -0.1; *p* = 0.523	B < 0.1; *p* = 0.971
PPT upper-cervical, median (25^th^,75^th^) †			
LFEM: 357.0(260.8–510.4) kPa	Ref		
HFEM: 447.0(295.3–606.6) kPa	B < 0.1; *p* = 0.922	Ref	
CM 368.2(283.4–521.7) kPa	B = 0.1; *p* = 0.636	B = 0.1; *p* = 0.758	Ref
CONTROLS 451.7(343.9–630.0) kPa	B = 0.2; *p* = 0.011	B = 0.2; *p* = 0.090	B = 0.2; *p* = 0.238
PPT lower cervical, median (25^th^,75^th^) †			
LFEM: 419.1(256.3–605.8) kPa	Ref		
HFEM: 546.8(336.0–634.9) kPa	B = 0.1; *p* = 0.411	Ref	
CM: 477.4(350.3–599.1) kPa	B = 0.1; *p* = 0.361	B < 0.1; *p* = 0.921	Ref
CONTROLS 543.3(426.1–749.0) kPa	**B = 0.3;** ***p*** = 0.001*	B = 0.2; *p* = 0.113	B = 0.2; *p* = 0.184
Ictal phase (n = 114)	LFEM	HFEM	CM
PPT trigeminal, median (25^th^,75^th^) †			
LFEM: 161.3(120.7–312.7) kPa	Ref		
HFEM: 153.6(110.7–186.1) kPa	B = -0.1; *p* = 0.336	Ref	
CM: 123.8(92.8–191.1) kPa	B = -0.4; *p* = 0.012	B = -0.3; *p* = 0.068	Ref
CONTROLS: 237.6(180.4–293.3) kPa	B = 0.3; *p* = 0.033	**B = 0.4;** ***p*** = 0.001*	**B = 0.7;** ***p*** < 0.001*
WUR trigeminal, median (25^th^,75^th^) †			
LFEM: 2.0(0.5–3.5)	Ref		
HFEM: 3.0(2.0–4.5)	B = 0.1; *p* = 0.081	Ref	
CM: 3.0(1.0–5.0)	B = -0.1; *p* = 0.129	B < -0.1; *p* = 0.963	Ref
CONTROLS: 2.0(0.0–3.0)	B = -0.1; *p* = 0.240	**B = -0.2;** ***p*** = 0.001*	**B = -0.2;** ***p*** = 0.006*
PPT upper-cervical, median (25^th^,75^th^) †			
LFEM: 325.3(276.2–598.4) kPa	Ref		
HFEM: 338.7(226.5–430.5) kPa	B = -0.2; *p* = 0.221	Ref	
CM: 269.9(205.6–457.4) kPa	B = -0.5; *p* = 0.012	B = -0.3; *p* = 0.113	Ref
CONTROLS: 451.7(343.9–630.0) kPa	B = 0.2; *p* = 0.173	**B = 0.4;** ***p*** = 0.006*	**B = 0.6;** ***p*** < 0.001*
PPT lower cervical, median (25^th^,75^th^) †			
LFEM: 437.5(298.6–697.2) kPa	Ref		
HFEM: 303.0(275.6–466.3) kPa	B = -0.2; *p* = 0.285	Ref	
CM: 297.1(213.1–511.9) kPa	B = -0.5; *p* = 0.010	B = -0.3; *p* = 0.072	Ref
CONTROLS: 543.3(426.1–749.0) kPa	B = 0.2; *p* = 0.143	**B = 0.4;** ***p*** = 0.007*	**B = 0.6;** ***p*** < 0.001*

CM: chronic migraine; g = gram; HFEM: high-frequency episodic migraine; LFEM: low-frequency episodic migraine; PPT: pressure pain threshold; Ref = reference level; WUR: Wind-up ratio; kPa: kilopascal;

Descriptive statistic was reported as median (25^th^, 75^th^)

^†^ = data were log-transformed for statistical analysis;

^*^significant at a p-value of 0.008 (Significant results were also boldly written)

**Table 3:** Linear regression model using QST variable as the dependent variable, group as predictor and gender, age, body mass index, use of preventive pharmacological therapy, and use of acute treatment in the 24 h before the evaluation as covariate in preictal and postictal phases

Preictal phase (116)	LFEM	HFEM
PPT trigeminal, median (25^th^,75^th^) †		
LFEM: 189.9(129.8–271.1) kPa	Ref	
HFEM: 197.3(150.9–238.2) kPa	B < -0.1, *p* = 0.780	Ref
CONTROLS 237.6(180.4–293.3) kPa	B = 0.2, *p* = 0.022	**B = 0.2,** ***p*** = 0.013*
WUR trigemina, median (25^th^,75^th^)l †		
LFEM: 2.0(0.3–5.0)	Ref	
HFEM: 2.0(0.0–3.0)	B = -0.1, *p* = 0.160	Ref
CONTROLS 2.0(0.0–3.0)	B = -0.1, *p* = 0.086	B < -0.1, *p* = 0.707
PPT upper cervical, median (25^th^,75^th^) †		
LFEM: 363.0(290.6–500.1) kPa	Ref	
HFEM: 383.8(294.3–473.3) kPa	B < -0.1, *p* = 0.869	Ref
CONTROLS 451.7(343.9–630.0) kPa	**B = 0.2,** ***p*** = 0.007*	**B = 0.3,** ***p*** = 0.006*
PPT lower cervical, median (25^th^,75^th^) †		
LFEM: 424.9(293.5–535.2) kPa	Ref	
HFEM: 481.0(334.6–571.8) kPa	B < 0.1, *p* = 0.970	Ref
CONTROLS 543.3(426.1–749.0) kPa	**B = 0.3,** p = 0.003*	**B = 0.3,** p = 0.005*
Postictal phase (n = 97)	LFEM	HFEM
PPT trigeminal, median (25^th^,75^th^) †		
LFEM: 165.3(137.8–211.8) kPa	Ref	
HFEM: 190.6(133.7–224.2) kPa	B = -0.1, *p* = 0.598	Ref
CONTROLS 237.6(180.4–293.3) kPa	B = 0.3, *p* = 0.022	**B = 0.3,** ***p*** = 0.005*
WUR trigemina, median (25^th^,75^th^)l †		
LFEM: 3.0(1.0–4.0)	Ref	
HFEM: 2.0(1.0–3.0)	B = -0.1, *p* = 0.273	Ref
CONTROLS 2.0(0.0–3.0)	B = -0.1, *p* = 0.031	B = -0.1, *p* = 0.339
PPT upper cervical, median (25^th^,75^th^) †		
LFEM: 339.2(280.8–497.0) kPa	Ref	
HFEM: 382.6(276.8–465.8) kPa	B < 0.1, *p* = 0.841	Ref
CONTROLS 451.7(343.9–630.0) kPa	**B = 0.3,** ***p*** = 0.003*	**B = 0.3,** ***p*** = 0.007*
PPT lower cervical, median (25^th^,75^th^) †		
LFEM: 412.7(306.4–507.6) kPa	Ref	
HFEM: 403.0(326.8–502.1) kPa	B < 0.1, *p* = 0.883	Ref
CONTROLS 543.3(426.1–749.0) kPa	**B = 0.4,** ***p*** = 0.001*	**B = 0.4,** ***p*** = 0.001*

CM: chronic migraine; g = gram; HFEM: high-frequency episodic migraine; LFEM: low-frequency episodic migraine; PPT: pressure pain threshold; Ref = reference level; WUR: Wind-up ratio; kPa: kilopascal;

Descriptive statistic was reported as median (25^th^, 75^th^)

^†^ = data were log-transformed for statistical analysis;

^*^significant at a p-value of 0.017 (Significant results were also boldly written)

### LFEM vs controls

However, when compared to controls, LFEM had reduced trigeminal (*p* = 0.001), and lower-cervical (*p* = 0.001) PPTs, and no differences in upper-cervical PPT and in trigeminal WUR interictally (all, *p* > 0.011) (Fig. 2, Table 2).

In both the preictal and postictal phases, LFEM had reduced upper-cervical (*p* = 0.007 and *p* = 0.003, respectively), and lower-cervical (*p* = 0.003 and *p* = 0.001, respectively) PPTs, and no differences in trigeminal PPT and trigeminal WUR (all, *p* > 0.022) compared to controls (Fig. 2, Table 3).

In the ictal phase, LFEM had no differences in any QST variables compared to controls (all, *p* > 0.033) (Fig. 2, Table 2).

### HFEM and CM vs controls

Interictally, HFEM and CM had no differences in any QST compared to controls (HFEM, all, *p* > 0.090; CM all, *p* > 0.103) (Fig. 2, Table 2).

On the other hand, in both the preictal and postictal phases HFEM had reduced trigeminal (*p* = 0.013 and *p* = 0.005, respectively), upper-cervical (*p* = 0.006 and *p* = 0.007, respectively), and lower-cervical (*p* = 0.005 and *p* = 0.001, respectively) PPTs, and no differences in trigeminal WUR (*p* = 0.707 and *p* = 0.339, respectively) compared to controls (Fig. 2, Table 3).

In the ictal phase, both HFEM and CM had reduced trigeminal (HFEM *p* = 0.001, CM *p* < 0.001), upper-cervical (HFEM *p* = 0.006, CM *p* < 0.001), and lower-cervical (HFEM *p* = 0.007, CM *p* < 0.001) PPTs, and higher trigeminal WUR (HFEM *p* = 0.001, CM *p* = 0.006) compared to controls (Fig. 2, Table 2).

### Correlations

In CM patients, the time from the last headache attack was significantly correlated with trigeminal PPT (r = 0.39, *p* = 0.029) and WUR (r = -0.38, *p* = 0.031) and with lower-cervical PPT (r = 0.36, *p* = 0.044). No significant correlation was observed with upper-cervical PPT (r = 0.30, *p* = 0.093). The time to the next headache attack was significantly correlated with trigeminal PPT (r = 0.36, *p* = 0.042) and WUR (r = -0.46, *p* = 0.008). No significant correlation was observed with upper-cervical (r = 0.26, *p* = 0.148), and lower-cervical (r = 0.30, *p* = 0.091) PPTs (Table 3).

In HFEM (ictal, postictal, and interictal phase), the time from the last headache attack was significant correlated with trigeminal (r = 0.45, *p* = 0.001), upper-cervical (r = 0.34, *p* = 0.018), and lower-cervical (r = 0.40, *p* = 0.006) PPTs and with trigeminal WUR (r = -0.39, *p* = 0.006) (Table 3).

In HFEM (ictal, preictal, and interictal phase), the time to the next headache attack was significant correlated with trigeminal (r = 0.43, *p* = 0.001), upper-cervical (r = 0.35, *p* = 0.009), and lower-cervical (r = 0.37, *p* = 0.005) PPTs and with trigeminal WUR (r = -0.44, *p* = 0.006). (Table 3).

In LFEM, no significant correlation was observed between the time from the last or the next headache attack and QST variables (Table 3).

## Discussion

This study suggests that the experimentally assessed pain sensitivity does not differ among LFEM, HFEM, and CM across the different phases of the migraine cycle [11–13]. However, HFEM and CM but not LFEM showed higher trigeminocervical pain sensitivity than healthy controls when assessed in the ictal phase but returned to the controls level during the interictal phase. For LFEM patients the opposite pattern was found, with higher trigeminocervical pain sensitivity than healthy controls during the interictal phase and then returned to the control level in the ictal phase. Moreover, in HFEM and CM, but not LFEM, the higher the pain sensitivity, the lower the interval from the last, or to the next headache attack. Thus, HFEM patients seem to have a sensory profile more like CM than LFEM. Furthermore, the study highlights the importance when pain sensitivity (QST) is assessed during the headache cycle and thereby explained the inconsistencies in the literature.

### Different sensory profile between LFEM and HFEM

Reduced pain threshold in the trigeminal and cervical areas and increased temporal summation of pain in the trigeminal area, could be considered a sign of pain sensitization in the trigeminocervical complex [18, 21]. To the author’s knowledge, this is the first comprehensive migraine study assessing the difference in pain sensitivity of the trigeminal area between LFEM and HFEM and how this changes differently across the migraine cycle between these subgroups.

Even though we found no differences between LFEM and HFEM in any phase of the migraine cycle, when compared to healthy subjects, LFEM showed significantly enhanced pain sensitivity of the trigeminocervical complex interictally [10, 22] which was not seen for HFEM. The lack of interictal pain sensitization in HFEM is in contrast with the previous literature [10, 22]. However, many QST studies did not exclude preictal patients who could drive the increased sensitization [10, 22].

On the contrary, during the ictal phase, HFEM had significantly enhanced pain sensitivity of the trigeminocervical complex [14, 18, 23, 24], while LFEM did not. This is the first study observing a reduction in the sensitization of the trigeminocervical area during the ictal phase in LFEM, and these results differed from the previous literature [16, 25]. One of the main mechanisms underlying the initiation of a migraine attack seems to be a “dysfunction” of cortical and subcortical areas involved in pain modulation that could switch from being antinociceptive to pronociceptive[26, 27]. This could lead to increased ictal sensitization of the trigeminocervical complex and concomitant headache [28, 29]. However, a subgroup of LFEM patients presents an enhanced activation of the antinociceptive system with a concomitant reduction in trigeminal pain sensitivity immediately before a headache attack [30]. Thus, it is possible that in these patients a “functional” antinociceptive system could be activated by a migraine attack, leading to a transitory reduction in trigeminocervical pain sensitivity observed ictally. This endogenous descending inhibitory control could be impaired in HFEM and CM patients, leading to higher headache frequency and chronification. In humans, endogenous descending inhibitory control could be assessed with conditioned pain modulation (CPM), and future studies should investigate CPM during the various phases of the migraine cycle in patients with different headache frequencies. However, as more appropriately designed studies found an increased ictal sensitization of the trigeminocervical complex in LFEM [16] these results should be interpreted with caution. Moreover, the high variance in QST results observed in ictal LFEM, suggested that only a subgroup of patients may present this ictal normalization in pain sensitivity. Future studies should focus on identifying this subgroup of patients.

During the perictal phase (preictal and postictal) the trigeminocervical pain sensitivity was similar between LFEM and HFEM, with both groups showing significantly enhanced pain sensitivity of the trigeminocervical complex compared to healthy controls. These results suggested that the perictal phases act as transition phases. In LFEM patients, the transition would be from a status of “interictal enhanced sensitization” to “ictal normal sensitization”, while for HFEM from a status of “interictal normal sensitization” to “ictal enhanced sensitization”. As a significant negative correlation between increased trigeminocervical sensitization and time from the last or to the next headache attack was observed in HFEM but not in LFEM, this transition is more enhanced in HFEM than LFEM. These results could explain why studies including both groups showed a significant correlation between time to the next headache attack and pain sensitivity[18, 31], while studies that included only LFEM did not [32]. Considering that HFEM patients have less time between two consecutive migraine attacks, changes in sensitization mechanism could occur more rapidly in this subgroup of patients.

The opposite pattern in cyclic changes in pain sensitivity observed between LFEM and HFEM could explain why studies that assessed differences across migraine phases in pain sensitivity pooling together LFEM and HFEM found heterogenous results [25, 33].

### Similar sensory profile between HFEM and CM

Interestingly, HFEM showed a sensory profile similar to CM. Both groups had enhanced ictal sensitization of the trigeminocervical complex compared to healthy subjects that normalized in the interictal phase. They also showed a significant negative correlation between enhanced sensitization and time from the last or to the next headache attack. The similarity observed between these two subgroups could explain why studies comparing pain sensitization between episodic migraine and CM did not find significant differences [34, 35]. These results suggested that the ictal sensitization of the trigeminocervical complex is more pronounced in patients with higher migraine frequency [14, 18, 23, 24]. Thus, future studies aimed to understand the mechanisms underlying migraine chronification should study ictal changes in pain sensitization. The interictal reduction in sensitization of the trigeminocervical complex observed in CM explained why previous studies did not find signs of enhanced cervical pain sensitivity in these patients [22].

### Limitations

The population was recruited from specialized headache centers, and over half of the patients were excluded for age, concomitant pathologies, and concomitant diagnosis of other headache types. Thus, the external validity of these results should be interpreted with caution.

The blindness of the assessor could not be maintained for the entire evaluation of every patient. QST in the trigeminal area was only assessed from one side to reduce the assessment duration, leading to a loss of blinding in patients with unilateral headache on the non-dominant side. However, the assessor would be blinded regarding the headache frequency and phase.

Moreover, the present study did not allow to control for the interval from the beginning of the headache phase. As known the sensitization occurs within 1–2 h after the beginning of the headache phase [36], this could have potentially led to the inclusion of ictal migraine patients in which the sensitization had not begun yet.

Another limitation is that in this study no questionnaires to assess allodynia symptoms were used.

Finally, as the study does not have a within-subjects design, comparison between different phases in each migraine subgroup should be interpreted with caution, and longitudinal studies with a within-subjects design must replicate these results Table 4.

**Table 4:** Spearman correlations analysis between QST results and time relative to the last or the next migraine attack in LFEM, HFEM and CM

	LFEM		HFEM		CM	
	Time from the last attack (interictal, ictal, postictal LFEM = 72)	Time to the next attack (interictal, ictal, preictal LFEM = 84)	Time from the last attack (interictal, ictal, postictal HFEM = 47)	Time to the next attack (interictal, ictal, preictal HFEM = 54)	Time from the last attack (ictal, interictal CM = 32)	Time to the next attack (ictal, interictal CM = 32)
PPT temporalis	r = 0.04 *p* = 0.772	r = 0.01 *p* = 0.957	**r = 0.45** ***p*** = 0.001*	**r = 0.43** ***p*** = 0.001*	**r = 0.39** ***p*** = 0.029*	**r = 0.36** ***p*** = 0.042*
WUR temporalis	r = -0.05 *p* = 0.708	r = -0.01 *p* = 0.965	**r = -0.39** ***p*** = 0.006*	**r = -0.44** ***p*** = 0.006*	**r = -0.40** ***p*** = 0.031*	**r = -0.46** ***p*** = 0.008*
PPT upper cervical	r = -0.01 *p* = 0.965	r = 0.03 *p* = 0.806	**r = 0.34** ***p*** = 0.018*	**r = 0.35** ***p*** = 0.009*	r = 0.31 *p* = 0.088	r = 0.26 *p* = 0.148
PPT lower cervical	r = -0.06 *p* = 0.622	r < 0.01 *p* = 0.993	**r = 0.40** ***p*** = 0.006*	**r = 0.37** ***p*** = 0.005*	**r = 0.36** ***p*** = 0.044*	r = 0.30 *p* = 0.091

CM: chronic migraine; HFEM: high frequency episodic migraine; LFEM: low frequency episodic migraine; PPT: pressure pain threshold; WUR: Wind-up ratio

^*^Spearman correlation significant at *p* < 0.05;

## Conclusion

The study showed that HFEM patients seem to have a QST profile matching CM better than LFEM, supporting the previous suggestion [5] to include HFEM in a revised diagnostic criterion for CM. Both HFEM and CM showed increased pain sensitivity in the trigeminocervical area during the ictal phase that normalized in the interictal phase. Both HFEM and CM showed a negative correlation between pressure pain sensitivity and duration from/to a headache attack, and a negative correlation between WUR and duration from/to a headache attack. LFEM showed increased pressure pain sensitivity in the trigeminocervical region during the interictal phase that normalized in the ictal phase. When assessing pain sensitivity in migraine populations the timing with respect to the headache phases is of utmost importance and can explain the inconsistency in pain sensitivity data reported in the literature.

## Supplementary Information

Below is the link to the electronic supplementary material.

Supplementary file1 (DOC 91 KB)
